# Neonicotinoid insecticide thiamethoxam compromises early larval development of the Amazonian tetra fish *Astyanax bimaculatus* (Linnaeus, 1758)

**DOI:** 10.1007/s10646-026-03115-7

**Published:** 2026-07-02

**Authors:** Bianca Lima de Sousa, Evagno Junior, Eduardo Albuquerque, Jeane Rodrigues Rodrigues, Sara Pedrosa, Vanessa Coimbra, Emanuelly Rodrigues, Caio Maximino, Diógenes Siqueira-Silva

**Affiliations:** 1 Research Group of Studies on the Reproduction of Amazon Fish (GERPA/LANEC), Biology Faculty (FACBIO), Federal University of South and Southern of Pará (Unifesspa), Av. dos Ipês, S/N, Marabá, 68507-590 PA Brazil; 2 Graduate Program in Animal Reproduction in the Amazon (ReproAmazon), Federal Rural University of the Amazon (Ufra) and Federal University of Pará (UFPA), Av. Presidente Tancredo Neves, Nº 2501, Terra Firme, 66, Belém, 077-830 PA Brazil

**Keywords:** Acute exposure, Early life stages, Ecotoxicology, LC50, Larval toxicity

## Abstract

Thiamethoxam (TMX) is a widely used neonicotinoid insecticide with high water solubility, which increases the likelihood of contamination in freshwater systems. Here, we assessed lethal and sublethal effects of TMX on the early life stages of the Neotropical Amazonian fish *Astyanax bimaculatus.* Two assays were performed: (i) embryonic exposure under static conditions (0, 0.05, 0.5, 5, and 50 mg/L; 200 embryos per treatment) and (ii) 96-h acute toxicity test on 1-day post-hatch larvae under semi-static conditions (0, 5, 25, and 50 mg/L; three aquaria of 100 larvae per treatment). During embryonic exposure, a visible coating over the chorion was observed at 5 and 50 mg/L, coinciding with both reduced normal hatching and increased larval deformities. The proportion of normally hatched larvae decreased from 97.5% in the control to 60.0% at 50 mg/L, while deformities increased to 15.5%, and unhatched embryos reached 24.5%. Post-hatch morphometry showed significant reductions in total length and yolk sac dimensions at concentrations ≥ 0.5 mg/L. In the larval assay, mortality increased in a dose-dependent manner, reaching mean values of 22.7%, 32.3%, and 64.0% at 5, 25, and 50 mg/L, respectively; the 96 h LC50 was 35.63 mg/L (95% CI: 27.83–43.44 mg/L). Overall, TMX impaired the survival and early development of *A. bimaculatus*, supporting the inclusion of early-life stage endpoints in the monitoring and risk assessment of Amazonian freshwater environments.

## Introduction

The intensive use of pesticides in agriculture is a primary driver of aquatic contamination (Kumar et al. 2023). These compounds enter rivers, lakes, and reservoirs via surface runoff, leaching, and atmospheric deposition (Albou et al. 2024). Once present in inland waters, pesticides pose severe risks to non-target organisms. In fish, exposure can cause lethal and sublethal effects that compromise the development, behavior, and survival of natural populations (Sabra and Mehana 2015).

In Brazil, the high commercialization of pesticides and rapid agricultural expansion have increased concerns over freshwater contamination, especially in sensitive regions like the Amazon (Belchior et al. 2017). The region’s high hydrological connectivity, combined with diffuse contaminant transport and accidental runoff events (ANA 2024), increases the exposure risk for aquatic organisms. This reinforces the need for ecotoxicological studies using native species (ANA 2025).

Neonicotinoids are of particular concern due to their agronomic efficiency and frequent detection in aquatic environments (Goulson 2013; Morrissey et al. 2015). They act as agonists of nicotinic acetylcholine receptors, causing hyperstimulation of the nervous system in target organisms (Tomizawa and Casida 2005). Their continuous occurrence in aquatic systems has been reported across different regions, supporting the need for ecotoxicological evaluations with aquatic organisms (Hladik et al. 2018). Beyond direct toxicity, continuous neonicotinoid inputs may disrupt aquatic food webs by reducing invertebrate prey, ultimately impacting fish populations (Yamamuro et al. 2019).

Early life stages of fish are highly vulnerable to contaminants because they involve intense cellular differentiation and developing physiological systems (Sant and Timme-Laragy 2018). Effects during this period may lead to mortality, delayed development, and morphological deformities (Jezierska et al. 2009). Furthermore, early exposure may impair critical behaviors, such as locomotion and anti-predator responses, reducing survival in the wild even when mortality is not immediate (Faria et al. 2020; Malhotra et al. 2021). Even minor disturbances during early development can impair recruitment, leading to long-term consequences for population dynamics (Chambers and Trippel 2012).

Thiamethoxam (TMX) is a major neonicotinoid characterized by high water solubility, which favors its persistence and mobility in surface and groundwater (Sánchez-Bayo et al. 2016; University of Hertfordshire, 2025). Research indicates that TMX impairs survival, hatching, and behavior in fish, especially during early life stages (Finnegan et al. 2017; Victoria et al. 2022a, b; Hasan et al. 2022). Exposure to neonicotinoid-contaminated river water has also been linked to altered gene expression in threatened freshwater species, reinforcing concerns about impacts during sensitive windows (Marchand et al. 2022). Some studies also report oxidative stress-related effects under thiamethoxam exposure, which may contribute to developmental impairment during early stages (Yan et al. 2016). Despite these risks, most evidence is derived from model species, leaving a critical data gap for native fish.

*Astyanax bimaculatus* is a native species widely distributed across the Amazon basin. Its ecological relevance and reproductive traits, including high fecundity (Rodrigues et al. 1992), fractional spawning (Cordeiro et al. 2020), and a well-described embryonic development (Weber et al. 2013), make it an ideal candidate for experimental toxicology. Given that TMX remains one of the most frequently detected neonicotinoids globally (Liao et al. 2024; Yang et al. 2025), this study evaluated the lethal and sublethal effects of TMX exposure during the embryonic and early larval stages of *A. bimaculatus*.

## Materials and methods

### Ethical considerations

All experimental procedures followed the guidelines of the Brazilian National Council for the Control of Animal Experimentation (CONCEA) and were approved by the Ethics Committee on Animal Use (CEUA) of the Federal University of the South and Southeast of Pará (Unifesspa) (protocol No. 23479.010692/2021-16). The project was also registered in the National System for the Management of Genetic Heritage and Associated Traditional Knowledge (SISGEN; No. A7BCB56).

### Animal acclimation

The study was conducted at the Laboratory of Neuroscience and Behavior (LANEC), Federal University of the South and Southeast of Pará (Unifesspa). Adult *A. bimaculatus* breeders were obtained from the laboratory facility and maintained in 100-L tanks under controlled conditions. Water quality was monitored daily and remained within reference values for fish culture: pH 7.09 ± 0.16 (Hanna Instruments^®^ pH meter), temperature 25.73 ± 0.80 °C (digital thermometer), and dissolved oxygen 9.30 ± 0.51 mg/L (Labcon Test). The photoperiod was set to a 12-h light : 12-h dark cycle. Fish were fed twice daily to apparent satiety with a commercial diet (50% crude protein and 4.6% fat; Acqua 243 line^®^).

### Breeding and fertilization

Twenty-four adult *A. bimaculatus* were distributed into two 13-L aquaria at a ratio of five males to seven females per tank. For induced breeding, fish were anesthetized using 2.5 mL of a eugenol solution (20 mL eugenol diluted in 180 mL absolute ethanol; Biodinâmica) diluted in 600 mL of water, following Rodrigues et al. (2023). Broodstock were then hormonally induced with crude carp pituitary extract (CCPE) diluted in 0.9% NaCl, adapted from Yasui et al. (2020). Females received two 0.1-mL doses via intraperitoneal injection below the pectoral fin at a 12-h interval. Males received a single 0.05-mL dose at the time of the second female injection. Following treatment, fish were held in 13-L tanks with constant aeration.

Six hours post-injection, semen was collected using a pipette and stored in 1.5-mL microtubes (Eppendorf) on ice (0 °C). Oocytes were obtained by gentle abdominal pressure in the anteroposterior direction and placed in PVC-lined Petri dishes. A pooled sample of oocytes was fertilized using pooled semen and activated with aquarium water. Following spawning, all broodstock were returned to their respective rearing tanks.

### Test chemical and solution preparation

Actara 250 WG^®^ (Syngenta Proteção de Cultivos LTDA, Brazil) was used. which is a commercial product containing 25% thiamethoxam (TMX) as the active ingredient. Stock and test solutions were prepared based on the following relationship: product mass (mg/L) = desired TMX mass (mg/L) ÷ 0.25.

### Embryonic exposure (preliminary concentration-screening test)

*Astyanax bimaculatus* embryos were collected immediately after fertilization to ensure uniform developmental stages. We performed a preliminary concentration-screening assay adapted from Hasan et al. (2022) to determine thiamethoxam (TMX) toxicity in newly fertilized embryos. The broad range of TMX concentrations was selected based on both environmentally relevant levels and data from previous toxicity studies. Exposure started at 0 h post-fertilization (hpf) and lasted until hatching.

**Fig. 1:**
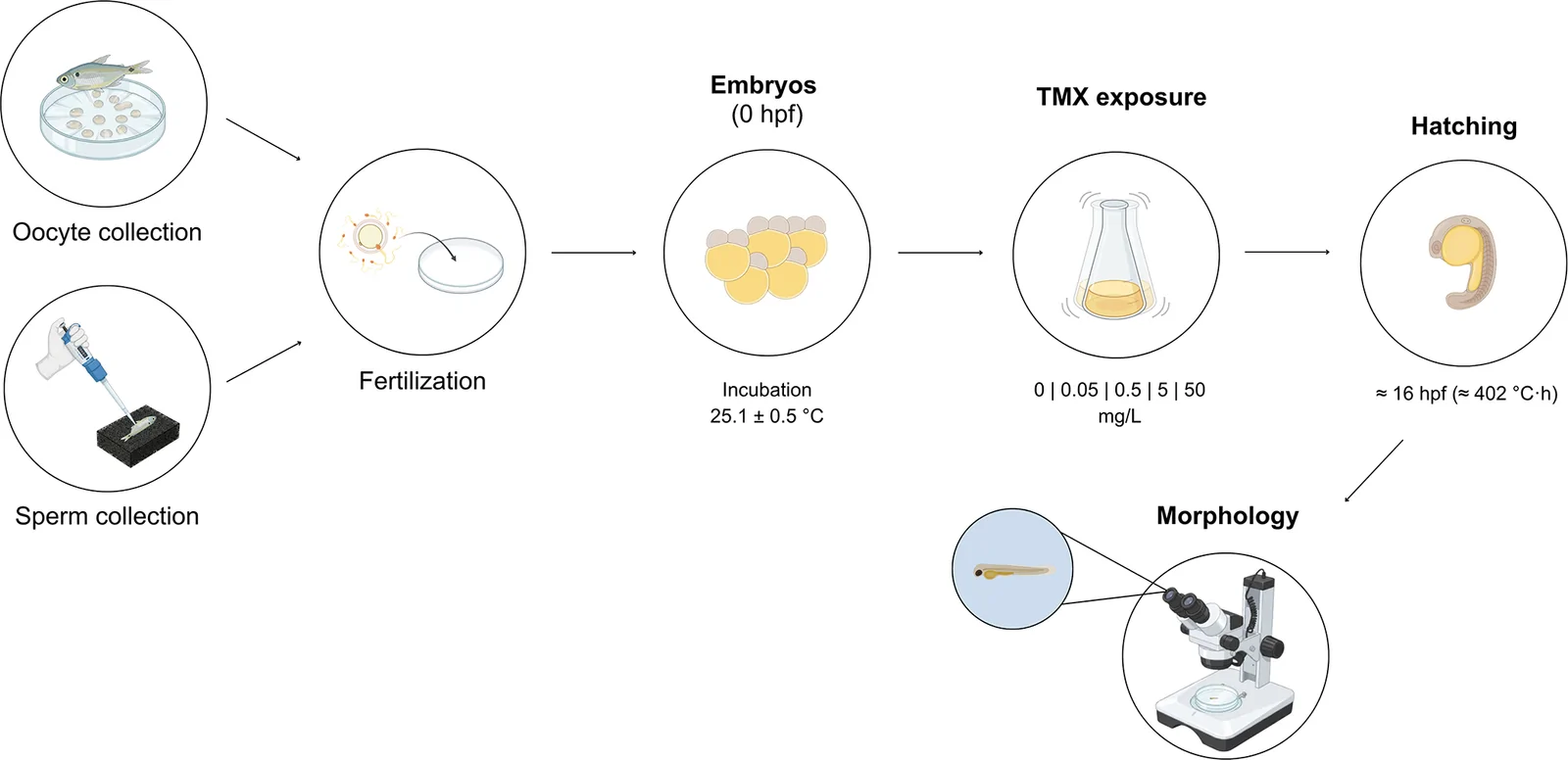
Experimental design of the embryonic exposure of *Astyanax bimaculatus* to thiamethoxam (TMX) from 0 h post-fertilization (hpf) until hatching, showing the tested nominal concentrations 0 (control) 0.05, 0.5, 5, and 50 mg/L

Embryos were exposed to nominal concentrations of the active ingredient thiamethoxam: 0 (control), 0.05, 0.5, 5.0, and 50 mg/L (Fig. 1). For each treatment, 200 embryos were placed in a single 1-L aquarium containing dechlorinated water under continuous aeration. A static system without water renewal was used. Test solutions were prepared directly in dechlorinated water. The water pH was adjusted to 7.0 by the controlled addition of NaOH or HCl (Merck^®^) and monitored daily. The incubation temperature was 25.1 ± 0.5 °C and was recorded hourly until hatching. Under these conditions, hatching occurred at approximately 16 hpf, corresponding to 402 degree-hours.

### Morphological and morphometric assessments

Immediately after hatching, larvae were euthanized with lethal dose of eugenol solution (20 mL eugenol in 180 mL 100% ethanol, BIODINÂMICA) and fixed in 2.5% glutaraldehyde in 0.2 M dibasic phosphate buffer (pH 7.4). Larval images were captured using a stereomicroscope and analyzed using ImageJ software (version 1.53). Morphometric measurements included total length and yolk sac volume (estimated as yolk length × yolk height). Fifteen larvae were randomly selected and measured per treatment. External morphological abnormalities, such as scoliosis, notochord malformations, and tail asymetry, were assessed under a stereomicroscope based on the criteria described by Pinheiro et al. (2021) for *Astyanax* species. Deformities were summarized descriptively as counts and percentages per treatment.

### Acute toxicity test in larvae (96 h)

**Fig. 2:**
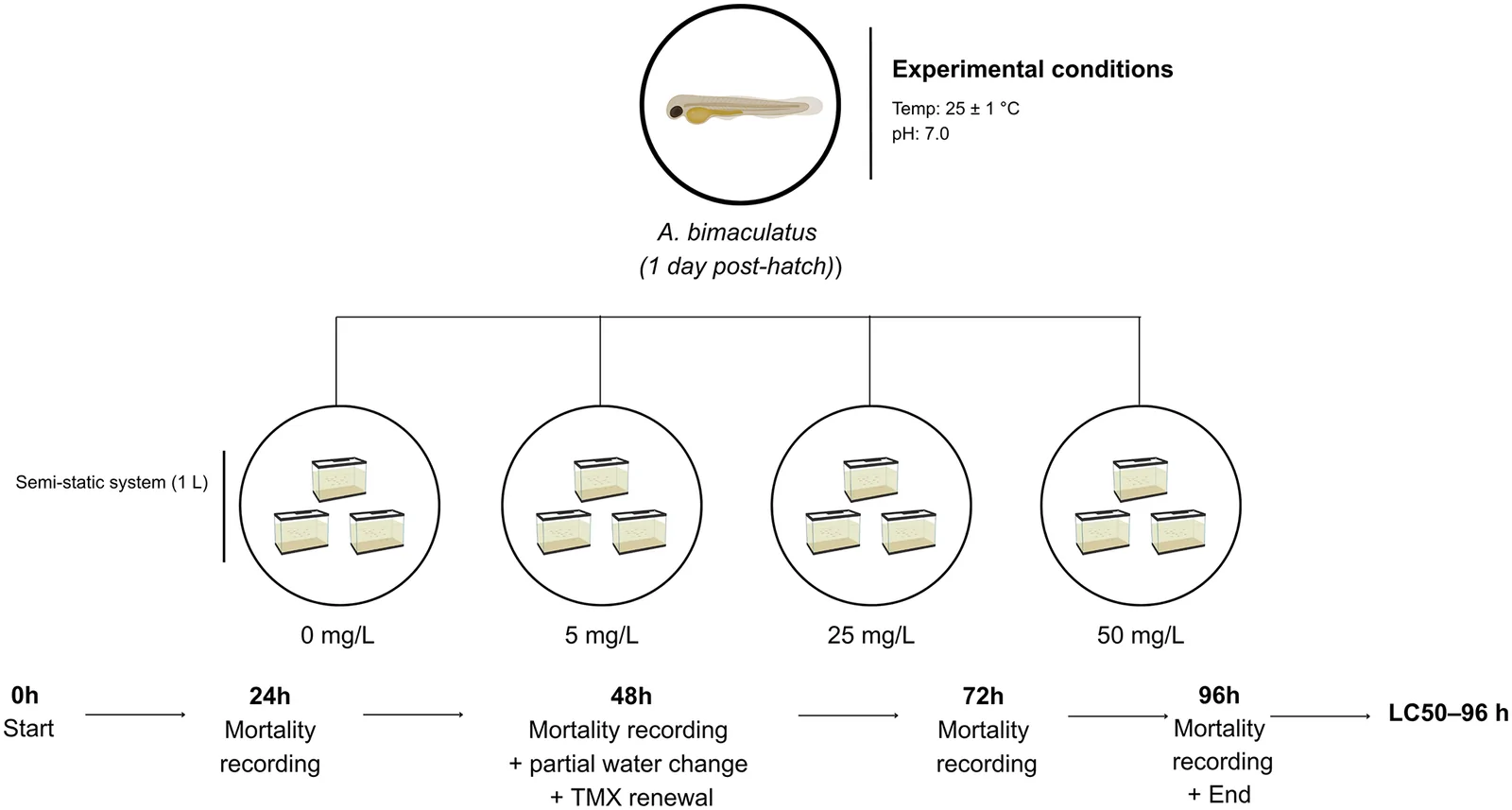
Experimental design of the acute toxicity assay using 1-day post-hatch (dph) *Astyanax bimaculatus* larvae exposed to thiamethoxam (TMX) at nominal concentrations of 0 (control), 5, 25, and 50 mg/L

An acute toxicity assay was performed to evaluate the effects of TMX on larvae *A. bimaculatus* at 1 day post-hatching (dph) and to estimate the 96-h LC50. Based on results of the preliminary embryonic screening, test concentrations were set at 0 (control), 5, 25, and 50 mg/L (Fig. 2). Each treatment was performed in triplicate, with 100 larvae per aquarium (*n* = 300 per concentration).

Larvae were maintained in 1-L aquaria under a semi-static system at 25 ± 1 °C with water pH adjusted to 7.0. Test solutions were prepared using dechlorinated water. Exposure conditions were maintained by a partial water renewal at 48 h. In accordance with OECD Test No. 203, larvae were not fed during the assay to prevent organic residue from compromising water quality. Mortality was recorded every 24 h up to 96 h. Larvae were considered dead if they failed to respond to gentle mechanical stimulation (OECD 2019). Dead individuals were removed immediately, and the resulting data were used to estimate 96-h LC50.

### Statistical analysis

The larval mortality dose-response was analyzed using a two-parameter log-logistic model (LL.2). The LC50 was estimated from cumulative mortality at 96 h with 95% confidence intervals using maximum likelihood estimation, including treatments with 0% or 100% mortality when applicable. For larval morphometrics, data normality was assessed using the Shapiro–Wilk test, and homogeneity of variances was evaluated using Levene’s test. Parametric data were analyzed by one-way ANOVA followed by Tukey’s HSD test for multiple comparisons. When parametric assumptions were not met, the Kruskal–Wallis test was applied, followed by Dunn’s test with Bonferroni correction. All statistical analyses were performed in R software (version 4.2.2), adopting a significance level of 5% (*p* < 0.05). In figures and tables, statistical significance was indicated by different letters for mortality data (post-hoc comparisons) and by an asterisk (*) for morphometric variables (*p* < 0.05).

## Results

### Chorion morphology during embryonic exposure

**Fig. 3:**
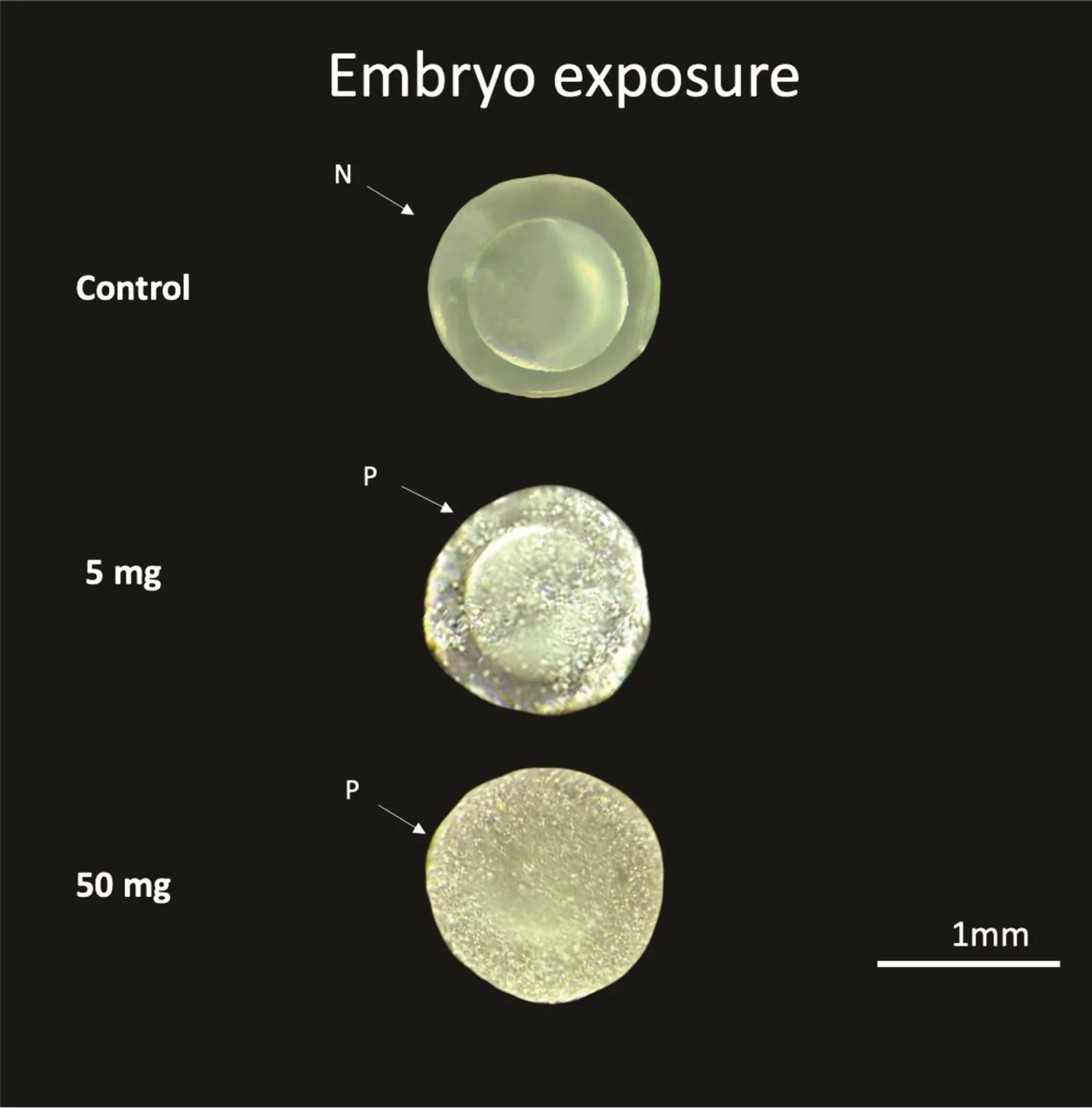
Developing *Astyanax bimaculatus* embryos during thiamethoxam exposure in the control (0), 5 mg/L, and 50 mg/L groups. Labels indicate a normal embryo (N) and the accumulation of pesticide residue over the chorion (P)

During embryonic development, a layer of adhered material was observed on the outer surface of the chorion at the highest TMX concentrations (5 and 50 mg/L), rendering the chorion opaque compared to the other treatments (Fig. 3). This accumulation was recorded in 100% of embryos exposed to 5 and 50 mg/L, whereas it was entirely absent in the control and lower concentration treatments (0.05 and 0.5). Due to this dense coating, it was not possible to visually monitor the embryonic developmental stages until prior to hatching. Consequently, some larvae in the highest concentration treatments also exhibited difficulty during the hatching process.

### Hatching and deformities after embryonic exposure

**Fig. 4:**
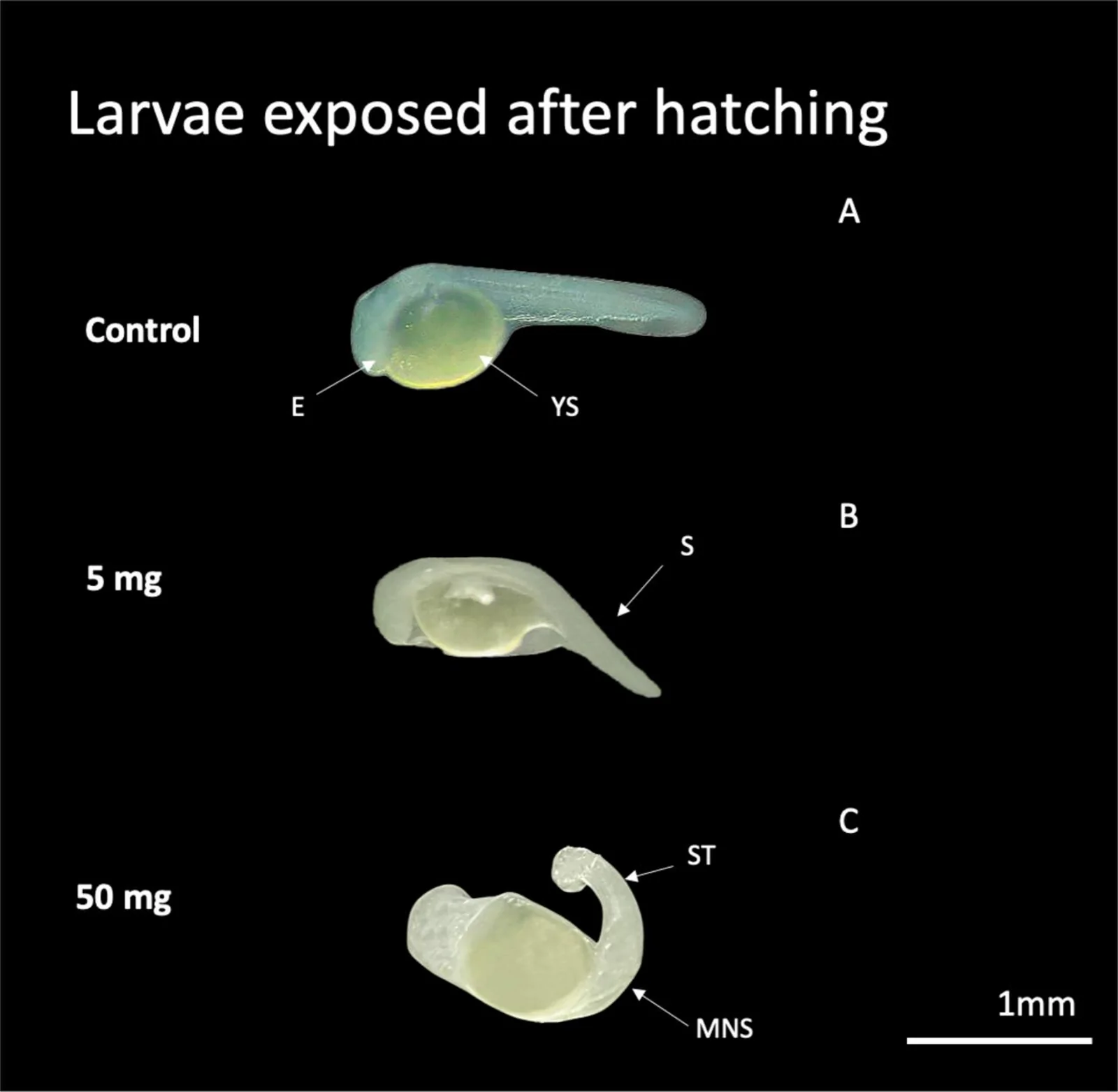
Morphological features of *Astyanax bimaculatus* larvae following embryonic exposure to different thiamethoxam concentrations. **A** Normal larvae: E, eye; YS, yolk sac; **B** Larvae exhibiting scoliosis (S); **C** Larva exhibiting a modified notochord structure (MNS) and a shortened tail (ST)

Embryonic exposure to TMX reduced the number of normally hatched larvae and increased the incidence of larval deformities, particularly at higher concentrations (Table 1). In the control group, 195 larvae hatched normally (97.5%), 2 exhibited abnormalities (1.0%), and 3 embryos failed to hatch (1.5%). In contrast, at the highest concentration of 50 mg/L, only120 larvae were normal (60.0%), 31 exhibited deformities (15.5%), and 49 embryos failed to hatch (24.5%). The observed morphological deformities included scoliosis, shortened tails, and structural alterations consistent with notochord malformation (Fig. 4).

**Table 1:** Number and percentage of normal and deformed *Astyanax bimaculatus* larvae obtained from embryos (*n* = 200 per treatment) exposed to different nominal concentrations of thiamethoxam (TMX) from 0 h post-fertilization (hpf) until hatching

TMX concentration (mg/L)	Initial embryos *n* (%)	Normal larvae *n* (%)	Deformed larvae *n* (%)	Unhatched embryos *n* (%)
0.00	200	195 (97.5)	2 (1.0)	3 (1.5)
0.05	200	191 (95.5)	3 (1.5)	6 (3.0)
0.50	200	185 (92.5)	3 (1.5)	12 (6.0)
5.00	200	160 (80.0)	7 (3.5)	33 (16.5)
50.0	200	120 (60.0)	31 (15.5)	49 (24.5)

### Post-hatch larval morphometry

After hatching, distinct morphometric differences were observed among treatments, with significant reductions in total length, yolk length, and yolk height in larvae exposed to 0.5, 5.0, and 50 mg/L (Table 2). Significant treatment effects were detected for total length (χ² = 55.61; *p* < 0.001), yolk length (χ² = 52.19; *p* < 0.001), and yolk height (χ² = 52.10; *p* < 0.001).

**Table 2:** Morphometric measurements (mean ± standard deviation) of post-hatch *Astyanax bimaculatus* larvae following embryonic exposure to different nominal concentrations of thiamethoxam (TMX)

Concentration (mg/L)	Yolk sac length (mm)	Yolk sac height (mm)	Total length (mm)	
0.00	0.863 ± 0.049	0.617 ± 0.036	2.968 ± 0.175	
0.05	0.865 ± 0.046	0.614 ± 0.034	2.867 ± 0.166	
0.50	0.770 ± 0.218*	0.537 ± 0.156*	2.595 ± 0.728*	
5.00	0.567 ± 0.043*	0.396 ± 0.027*	2.049 ± 0.089*	
50.0	0.543 ± 0.096*	0.382 ± 0.065*	1.722 ± 0.339*	

* statistically significant difference (*p* < 0.05)

### Acute larval mortality after 96 h of exposure

**Fig. 5:**
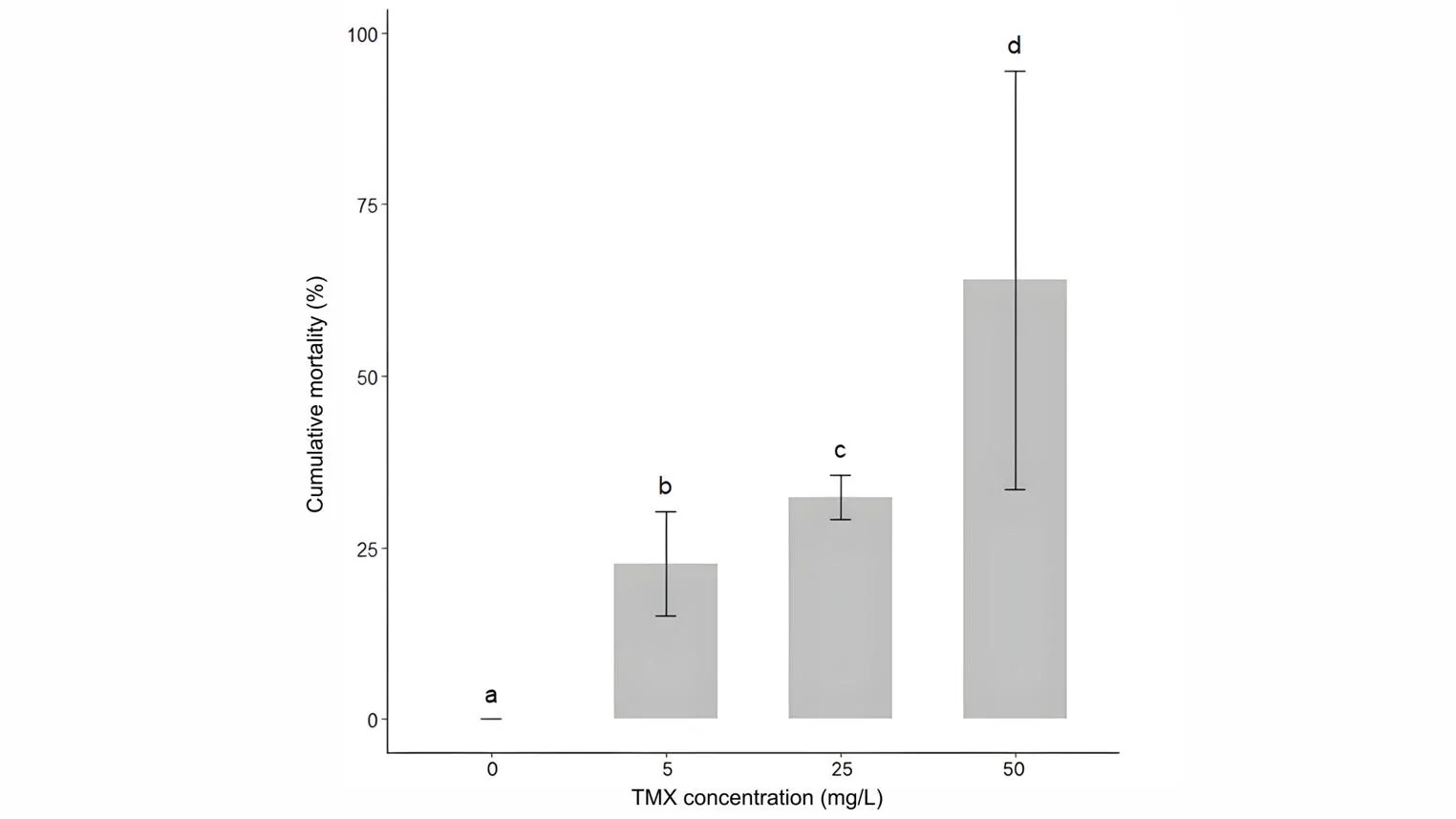
Cumulative mortality (%) of *Astyanax bimaculatus* larvae after 96 h exposure to thiamethoxam (TMX) at 0, 5, 25, and 50 mg/L. Bars represent mean ± standard deviation (*n* = 3 replicates per treatment). Different letters indicate significant differences among treatments (*p* < 0.05)

In the acute toxicity assay (96 h), TMX caused a clear concentration-dependent increase in larval mortality. No mortality occurred in the control group. Mean cumulative mortality reached 22.7% at 5 mg/L (95% CI: 18.3–27.7%), 32.3% at 25 mg/L (95% CI: 27.3–37.8%), and 64.0% at 50 mg/L (95% CI: 58.4–69.2%) (Fig. 5). Multiple comparisons revealed that mortality at 25 mg/L was significantly higher than at 5 mg/L (*p* = 0.041), while mortality at 50 mg/L was significantly higher than both 5 and 25 mg/L (*p* < 0.001 for both comparisons).

### larval LC50 (96 h) estimation

**Fig. 6:**
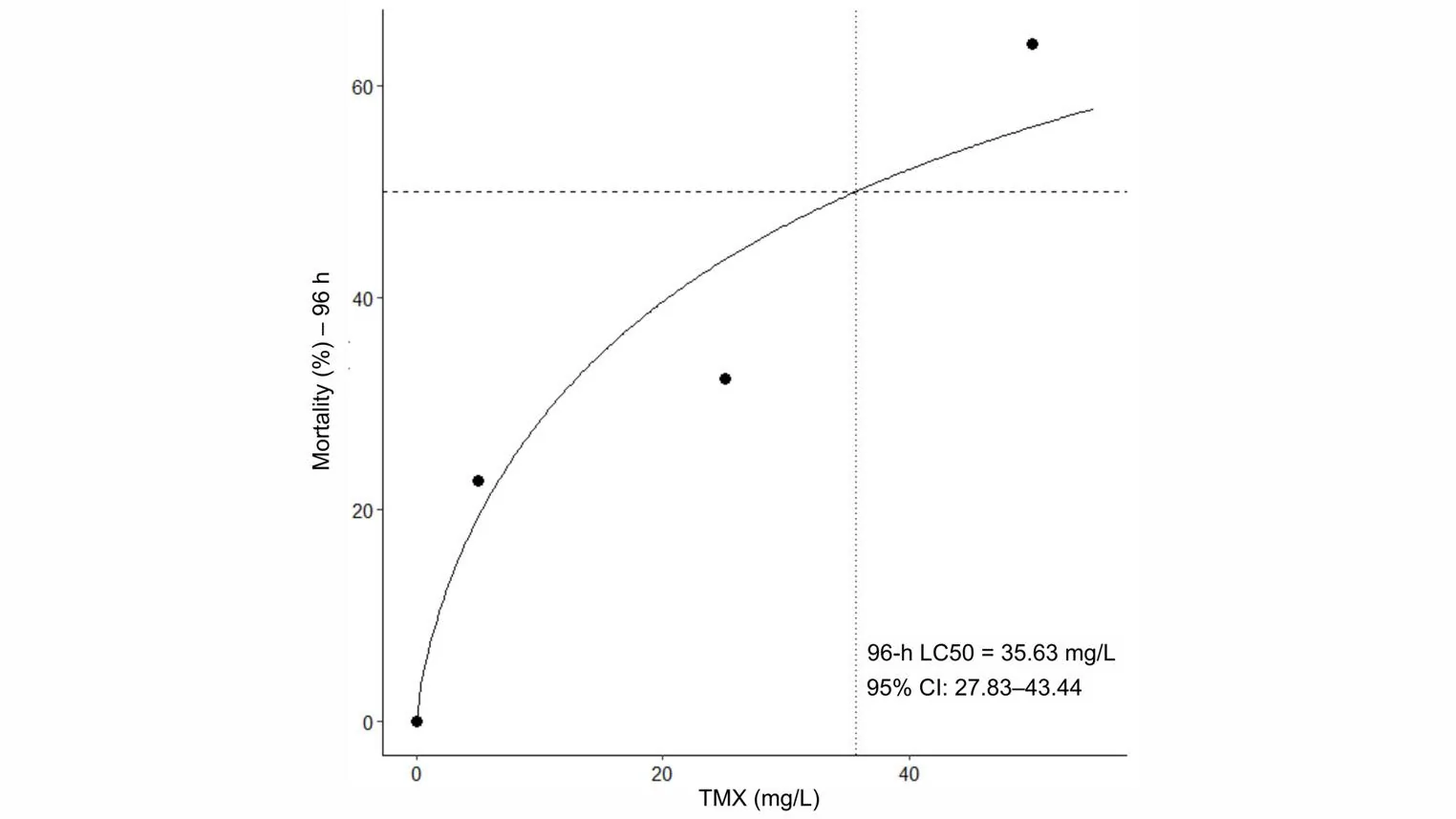
Concentration–response curve for 96-h larval mortality of *Astyanax bimaculatus* exposed to thiamethoxam. Points represent observed mean mortality per concentration, and the solid line indicates the fitted two-parameter log-logistic model. Horizontal and vertical dashed lines denote the 96-h LC50 value (35.63 mg/L) and its corresponding 95% confidence interval (27.83-3. Mg/L)

The two-parameter log-logistic model provided an adequate description of the concentration-response pattern and was used to estimate the median lethal concentration. The 96-h L50 was determined to be 35.63 mg/L (95% CI: 27.83–43.44 mg/L) **(**Fig. 6**)**, representing the concentration associated with 50% larval mortality under the tested conditions.

## Discussion

The concentration-dependent increase in both unhatched embryos and larval mortality observed in this study is consistent with biochemical and physiological findings in other teleost species, such as *Oreochromis niloticus* and *Catla catla*. In *O. niloticus*, TMX exposure alters biochemical parameters, leading to the degradation of cellular biomolecules and essential enzymes; these cellular-level disruptions ultimately culminate in the systemic failure of vital organs (Temiz and Kargin, 2024). Similarly, in the freshwater fish *C. catla*, TMX significantly alters biochemical profiles (including proteins, glucose, and electrolytes), inhibits gill Na+/K+ -ATPase activity, and disrupts both enzymatic and non-enzymatic antioxidant defenses, such as superoxide dismutase (SOD), catalase (CAT), lipid peroxidation (LPO), reduced glutathione (GSH), glutathione peroxidase (GPx) and glutathione S-transferase (GST) (Veedu et al. 2022).

The chorion coating observed at the highest TMX concentrations suggests that exposure directly affected the embryo surface. Because the chorion acts as a vital protective barrier, alterations to this structure can compromise fundamental physiological processes such as gas exchange. The adhered material likely obstructs chorion pores, thereby reducing oxygen availability; this induced hypoxia can impair development and hinder the hatching process (Ciuhandu et al. 2007; Duan et al. 2020), as observed in the highest concentration treatments. Consequently, and following the same general pattern reported for thiamethoxam and other neonicotinoids during early fish development (Hasan et al. 2022; Victoria et al. 2022a, b), elevated TMX concentrations reduced normal hatching rates, while increasing both the incidence of deformities and the number of unhatched embryos in *A. bimaculatus*.

The observed linear decline in both yolk volume and larval length in response to increasing TMX concentrations indicates a predictable toxicological trend that reflects a significant metabolic cost. Energy reserves normally allocated for somatic growth appear to have been diverted toward homeostatic maintenance and detoxification pathways. Furthermore, the simultaneous reduction in yolk volume and larval body length suggests that the larvae may be experiencing a stress-induced hypermetabolic state, causing them to deplete their endogenous energy reserves prematurely. Ecologically, smaller larvae face an elevated risk of predation, and a reduced yolk volume narrows the critical temporal window available to transition to exogenous feeding before starvation occurs in natural habitats. Similar morphological impairments and yolk-sac edemas have been documented in *Salmo salar* exposed to bisphenol A (Honkanem et al. 200), supporting the hypothesis that TMX disrupts normal development. Because neonicotinoids act as agonists on nicotinic acetylcholine receptors, the interference with nervous system function during early ontogeny provides a mechanistic explanation for these developmental anomalies (Tomizawa and Casida 2005). Acordingly, TMX exposure has been directed linked to neurotoxicity and impaired locomotor or behavioral performance in fish larvae (Li et al. 2025).

In the 96-h larval assay, mortality increased in a concentration-dependent manner, a classic hallmark of acute toxicity. However, relying solely on lethal endpoints may underestimate environmental risk; survival must be interpreted alongside sublethal biological shifts (Jeninga et al. 2023). Our results demonstrate that even at concentrations where mortality is low, *A. bimaculatus* suffers significant physiological costs, such as reduced yolk and stunted growth. These morphological impairments likely overlap with other systemic stressors documented in recent literature, including compromised immune-related pathways in fish larvae (Kooij et al. 2025) and potential transgenerational deficits that extend to subsequent generation (Zhang et al. 2023). Together, these data argue for a more holistic approach to ecological risk assessment, one that recognizes mortality as merely the final stage of a broader cascade of insecticide-induced damage.

The 96-h LC50 obtained here must also be viewed in the context of species-specific sensitivy and life-stage vulnerability. While reported TMX LC50 values for adult fish are often high, exceeding 125 mg/L for rainbow trout (*Oncorhynchus mykiss*) and 114 mg/L for bluegill (*Lepomis macrochirus*) (Qamar et al. 2023), early life stages demonstrate much higher sensitivity. For instance, in *Trichogaster fasciata* larvae (1 dph), the LC50 decreased over time, reaching a much lower threshold of 0.27 mg/L at 96 h (Hasan et al. 2022). It is critical to acknowledge that this wide interspecific variability is driven not only by intrinsic biological differences, such as metabolic rates and surface-area-to-volume ratios, but also by heterogeneous experimental conditions, including water hardness, temperature, and varying purity of commercial TMX formulations. Recognizing these confounding variables reinforces the importance of using sensitive early life stages of native species to accurately assess risks to recruitment and long-term population maintenance.

In conclusion, this study provides the first evidence that thiamethoxam exposure impairs the early development of the native Neotropical fish *A. bimaculatus*. The observed concentration-dependent reduction in yolk reserves and body length indicates a metabolic disruption that compromises larval fitness and potential survival in the wild. From an ecological risk assessment perspective, these results suggest that thiamethoxam poses a tangible threat to fish recruitment in Neotropical watersheds. Given the typical TMX concentrations reported in surface waters (0.05 to 0.5 µg/L), and our calculated LC50, the resulting *Risk Quotient (RQ = 0.14)* indicates that current environmental exposure levels pose a moderate risk to the early life stages of this native species. Crucially, because sublethal effects occurred at even lower concentrations, standard *RQ* frameworks may underestimate the true ecological pressure on wild fish recruitment. We propose that larval growth and yolk-sac bioenergetics be integrated into environmental monitoring programs as sensitive biomarks of pesticide impact. Furthermore, our findings advocate for a revision of current water quality criteria to better protect native biodiversity against the sublethal pressures of modern neonicotinoid use. While this study establishes the morphological and development benchmarks for thiamethoxam toxicity in *A. bimaculatus*, future studies focusing on the underlying enzymatic and molecular pathways are required to fully elucidate these mechanisms.

## Data Availability

No datasets were generated or analysed during the current study.
